# DFT Study on Intermetallic Pd–Cu Alloy with Cover Layer Pd as Efficient Catalyst for Oxygen Reduction Reaction

**DOI:** 10.3390/ma11010033

**Published:** 2017-12-26

**Authors:** Ji Liu, Xiaofeng Fan, Chang Q. Sun, Weiguang Zhu

**Affiliations:** 1 Centre for Micro-/Nano-Electronics (NOVITAS), School of Electrical and Electronic Engineering, Nanyang Technological University, 50 Nanyang Avenue, Singapore 639798, Singapore; JLIU024@e.ntu.edu.sg (J.L.); ECQSUN@ntu.edu.sg (C.Q.S.); 2 Key Laboratory of Automobile Materials, Ministry of Education, and College of Materials Science and Engineering, Jilin University, Changchun 130012, China

**Keywords:** ORR, intermetallic, first-principle calculations, surfaces

## Abstract

Detailed density functional theory (DFT) calculations of the adsorption energies (E_ad_) for oxygen on monolayer Pd on top of the Pd–Cu face-centered cubic (FCC) alloy and intermetallic B2 structure revealed a linear correspondence between the adsorption energies and the *d*-band center position. The calculated barrier (E_barrier_) for oxygen dissociation depends linearly on the reaction energy difference (ΔE). The O_2_ has a stronger adsorption strength and smaller barrier on the intermetallic Pd–Cu surface than on its FCC alloy surface. The room-temperature free energy (ΔG) analysis suggests the oxygen reduction reaction (ORR) pathways proceed by a direct dissociation mechanism instead of hydrogenation into OOH. These results might be of use in designing intermetallic Pd–Cu as ORR electrocatalysts.

## 1. Introduction

Proton exchange membrane fuel cells (PEMFCs) have attracted much attention over the last decades due to the advantages of high energy conversion efficiency and the clean energy system [1,2,3]. Although considerable advances have been achieved in the past years, the obstacles, such as the anode CO poison issue and sluggish kinetics of the cathode oxygen reduction reaction (ORR), have hindered the widespread applications of fuel cells [4,5,6]. Platinum (Pt) has the highest catalytic activity for ORR among pure metals. However, the high cost of Pt and its poor stability and durability are the major drawbacks for the commercialization of Pt-based fuel cells. Previous studies have pointed out that alloying Pt with transition metals can induce Pt–Pt bond compression, resulting in enhanced activity and stability [7,8,9,10,11]. Recently, developing non-Pt catalysts towards fuel cell applications has become a hot topic [12,13,14]. The potential catalysts must meet the standard of lower cost while exhibiting comparable or even better ORR activity than commercial Pt catalysts. Among the potential catalysts, palladium (Pd) and its alloys have been a popular choice due to the comparatively inexpensive cost and high ORR performance [13,15,16].

Theoretical calculations and experimental results have indicated that Pd–M alloys will undergo phase segregations upon annealing at elevated temperature, resulting in a pure Pd overlayer on the bulk alloys, where M represents transition metals [16,17]. Using the *d*-band model proposed by Nǿrskov et al., the enhanced catalytic activity is explained by the electronic and geometric influence of the alloying element, making the overlayer Pd atoms different from Pd atoms in the bulk [8,18,19,20]. However, the critical problem of this Pd alloy core/shell structure is that the core generally consists of random distributed atoms depending on Pd/M ratios, making it difficult to determine and control the active sites experimentally [11,21,22,23]. Poorer durability in acid than Pt is also one of the disadvantages limiting the application of Pd-based alloys as cathode materials. Recently, with the development of synthesizing techniques, the intermetallic alloy has been successfully prepared and investigated experimentally, and the intermetallic alloy has enhanced resistance to corrosion and better catalytic activity due to ordered long-range atom distribution [11,24,25,26,27,28,29,30,31]. For example, Pd–Cu can undergo the disorder-to-order transformation from FCC to a B2 ordered structure [32]; Pd–Fe can be converted from FCC to the face-centered tetragonal (FCT) ordered phase [5]. However, the understanding of the micro-mechanism is still not clear in detail. In particular, detailed research about the design rule for ORR based on intermetallic alloy is still lacking.

In this work, intermetallic Pd–Cu with monolayer Pd on supporting substrate Pd–Cu alloy (B2 structure) has been proposed and calculated by first principle calculations. It is noted that the monolayer model studied here is not exactly the experimentally synthesized core/shell nanoparticle after phase segregation. Here, with slab model, we focus on the surface reactivity of Pd–Cu alloy with atomic cover layer Pd. We have examined the density of states (DOS) of the compressed Pd monolayer on the intermetallic Pd–Cu and compared with that of FCC alloy Pd–Cu and bulk Pd. An obvious down-shift of the *d*-band center has been witnessed. In order to figure out the relationship between oxygen adsorption energy and ORR activity, the dynamic barrier of oxygen dissociation is explored. By calculating the room temperature free energy change (ΔG) with the analysis of reaction paths about ORR, we expect that intermetallic Pd–Cu can serve as alternate promising cathode materials.

## 2. Computational Details

All the calculations were performed on the basis of spin-polarized density functional theory (DFT) with projector augmented wave (PAW) formalism, as implemented in the Vienna ab initio simulation package (VASP) code [33,34]. The generalized gradient approximation with the parameterization of Predew–Burke–Ernzerh (PBE) was used for the exchange–correlation functional [35,36]. A kinetic energy cutoff of 400 eV for the plane wave expansion with proper *k*-point spacing using Monkhorst–Pack grid method [37] was found to be sufficient to ensure that the total energy was converged at 1 meV/atom level. The convergence criterion for the self-consistence field energy was set to be 10^−4^ eV. In this work, we have first built two corresponding unit cells (FCC alloy and B2 intermetallic) and calculated the lattice constants as listed in Table 1. The Pd/Cu ratio of intermetallic is 50/50, which is proven to be the maximum catalytic activity composition for ORR [38,39,40]. We have then adopted the same Pd/Cu ratio (50/50) for Pd–Cu FCC alloy. The lattice constants (a = b = c = 3.021 Å for intermetallic Pd–Cu B2 structure, a = b = c = 3.811 Å for Pd–Cu FCC alloy) are in accordance with previous experimental results and DFT calculations [41,42]. We have calculated the heats of formation (ΔH_f_) for both Pd–Cu B2 intermetallic and FCC alloy. The results show that Pd–Cu B2 structure (−13.42 KJ/mol) has lower heats of formation than that of FCC alloy (−10.93 KJ/mol).

**Figure 1:**
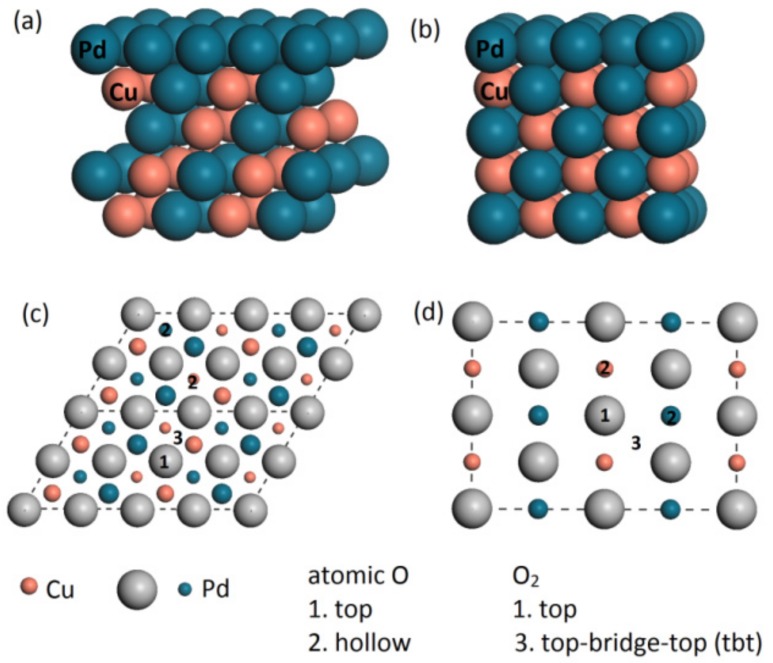
The configuration of side view of (**a**) Pd/Pd–Cu(111) alloy and (**b**) intermetallic Pd/Pd–Cu(110); and top view of (**c**) Pd/Pd–Cu(111) alloy and (**d**) intermetallic Pd/Pd–Cu(110). The atomic oxygen adsorption site, O_2_ adsorption site are shown in Figure 1c,d. Regardless of the atom size, orange color represents Cu and ashen and dark blue color represent Pd.

By comparing the ORR activity on these two models (Pd–Cu FCC alloy and B2 intermetallic), we can explore the possible application for intermetallic in fuel cells. When simulating the Pd monolayer on top of two different cores, we have used the slab method by building a pseudomorphic layer, where the lateral lattice spacing is the same as the core. After phase segregation, monolayer Pd is placed on top of four-layer Pd–Cu(111) (FCC) or Pd–Cu(110) (B2), depending on the unit cell structure. We have chosen (111) surface for FCC alloy and (110) surface for B2 alloy due to the low Miller index, good surface stability and reactivity. The vacuum region is up to 15 Å in order to avoid the spurious coupling effect in *z* direction. The bottom two layers are fixed with the value of bulk alloy in the structural relaxation. We have adopted (2 × 4) surface unit cells to represent the sample surface, with minimum coverage up to 1/8 ML as shown in Figure 1. For these models, we have used the following notations: Pd/Pd–Cu(111) represents monolayer Pd on top of Pd–Cu FCC alloy and Pd/Pd–Cu(110) represents monolayer Pd on top of Pd–Cu B2 intermetallic. For reference, we analyzed the adsorption on bulk Pd with five-layer Pd slab along [111] direction which is denoted as Pd(111) with (2 × 2) surface unit cell [43]. During the calculation, we have used 4 × 2 × 1 *k*-point mesh for Pd/Pd–Cu(111), 4 × 3 × 1 *k*-point mesh for Pd/Pd–Cu(110) and 5 × 5 × 1 *k*-point mesh for Pd(111), respectively. We can see that, due to lattice mismatch, the Pd monolayer is compressed with up to 3.49% to fit bulk Pd–Cu lattice for the Pd/Pd–Cu(111) system. Previous studies by our group and other studies have indicated that this compression effect can partly explain the change of DOS and *d*-band center, which is related to catalytic activity [10,28,43]. For reference, we have built and calculated a five-layer Pt(111) slab and Pt monolayer on top of four-layer PdCu(110), which are denoted as Pt(111) and Pt/Pd–Cu(110), respectively. The computational parameters (such as *k*-point mesh) are the same as Pd/Pd–Cu(110) for Pt/Pd–Cu(110) and the same as Pd(111) for Pt(111), respectively.

For the adsorption on the surface, the adsorption energy (E_ad_) is defined by the formula, E_ad_ = E_tot_ − E_slab_ − E_A_, where E_tot_, E_slab_, and E_A_ are the energies of the slab with adsorbate A, isolated slab for the clean metal surface, and isolated adsorbate A, respectively. From the definition, a negative value of E_ad_ corresponds to exothermic adsorption. The nudged elastic band (NEB) method was used to search the minimum energy path and determine the transition state (TS) for O_2_ dissociation [44].

## 3. Results and Discussion

### 3.1. The Adsorption of O_2_ on Pd/PdCu Surface

Before investigating the possible pathways for ORR, we have first studied the adsorption of atomic O and dissociation of O_2_ on Pd overlayer. The adsorption sites are illustrated in Figure 1c,d and the results for atomic O adsorption are listed in Table 2. It is clearly seen that hollow site is more favorable than top site, which is consistent with previous DFT studies [16,45]. There are two kinds of hollow sites: hollow_Pd (top view) and hollow_Cu (top view). Here, we have calculated the adsorption energies on both hollow sites, and the results show that the adsorption energies only have a negligible difference (about 0.1 eV). The adsorption energy for the top site is positive, indicating that the adsorption is endothermic. DFT calculations by Tang et al. indicates that Cu atoms can reduce the Pd–O binding energy [46]. From Table 2, it is clearly seen that, by alloying, the adsorption strength of oxygen is reduced compared with that of Pd(111) surface.

**Figure 2:**
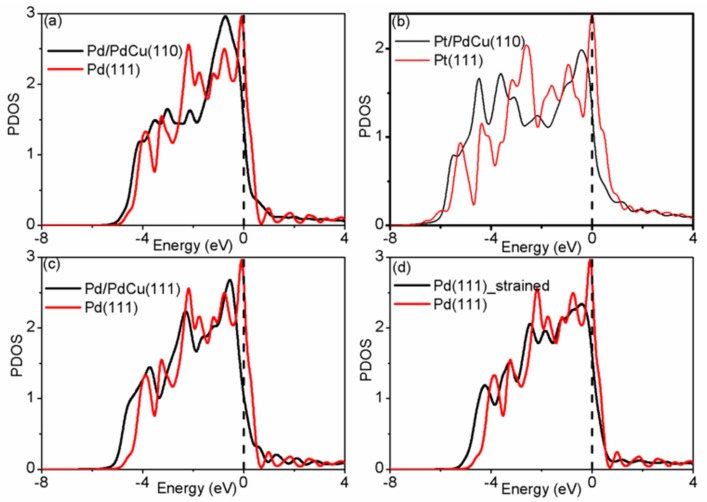
The calculated partial density of states (PDOS) for (**a**) Pd monolayer on top of intermetallic Pd–Cu; (**b**) Pt monolayer on top of intermetallic Pd–Cu; (**c**) Pd monolayer on top of Pd–Cu FCC alloy, and (**d**) compressed Pd(111). Noted that Pd/Pd–Cu(111) and compressed Pd(111) have the same strain up to 3.49%.

**Figure 3:**
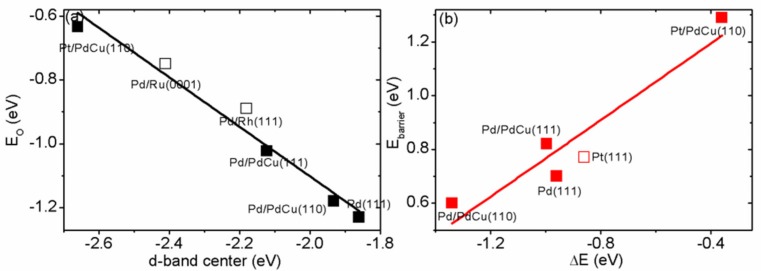
The calculated (**a**) adsorption energy of atomic O as a function of the *d*-band center of surface Pd relative to Fermi level (marked with black solid square) and (**b**) plot of Brǿnsted–Evans–Polanyi relations for O_2_ dissociation (marked with red solid square). The red solid line is the fitting line with the equation E_barrier_ = 0.714 × ΔE + 1.479. The added data in Figure 3a (marked with black empty square) are from Reference [16] and the added date in Figure 3b (marked with red empty square) is from Reference [45], respectively.

In order to understand the adsorption trend and details of bond formation on the surface, we have analyzed the electronic properties by calculating the density of states (DOS). The partial DOS (PDOS) is shown in Figure 2. Due to phase transformation, the PDOS shape of Pd monolayer on intermetallic Pd–Cu (B2 structure) is different from that of FCC Pd overlayer. The localized DOS near the Fermi level of surface Pd overlayer are considered to be related to the adsorption strength. Pt monolayer on intermetallic Pd–Cu has the least localized DOS near the Fermi level and Pd monolayer on intermetallic Pd–Cu has the highest localized DOS. This sequence is in good agreement with the adsorption trend. In order to study the strain effect, we have built and calculated a compressed Pd(111) with five-layer slab along [111] direction, which has the same stain as Pd/Pd–Cu(111). From Figure 2c,d, we can clearly see that the DOS are modified by both strain effect due to lattice mismatch and charge transfer between the substrate and Pd monolayer. In order to understand the charge transfer between the substrate and Pd monolayer, we have estimated the electron transfer by calculating the difference of number of electrons for Pd monolayer with and without the substrate for Pd/Pd–Cu(111) and compressed Pd(111). This is obtained by integrating the DOS of Pd monolayer. For compressed Pd(111), the estimated electron transfer from substrate to Pd surface is 0.22 electron per atom; for Pd/Pd–Cu(111), the estimated electron transfer from substrate to Pd monolayer is 0.33 electron per atom. Upon alloying, the positions of *d*-band center (*ε_d_*) shift downwards (away from the Fermi level). This downward shifting is related to the change of adsorption energy on various Pd surfaces. As shown in Table 2, the *d*-band center of Pd(111) is −1.86 eV, which is supported by previous reports [47]. The calculated *d*-band center for compressed Pd(111) is −2.02 eV, which is located between Pd(111) (−1.86 eV) and Pd/Pd(111) (−2.12 eV). This is in accordance with the corresponding trend of localized PDOS near Fermi level and the estimated electron transfer from the substrate. Previous DFT calculations have revealed that insertion of Cu atoms in Pd lattice affects geometric and electronic properties of Pd [48]. An obviously linear relationship between *d*-band center (*ε_d_*) and atomic oxygen adsorption energy (E_O_) is witnessed and shown in Figure 3a, where we have added some data from other studies for better understanding [16,45]. This linear relationship is supported by *d*-band center model proposed by Nǿrskov et al., which is widely applied in understanding the bond formation and activity trend among transition metals [49,50].

### 3.2. The Dissociation of O_2_ on Pd/PdCu Surface

**Figure 4:**
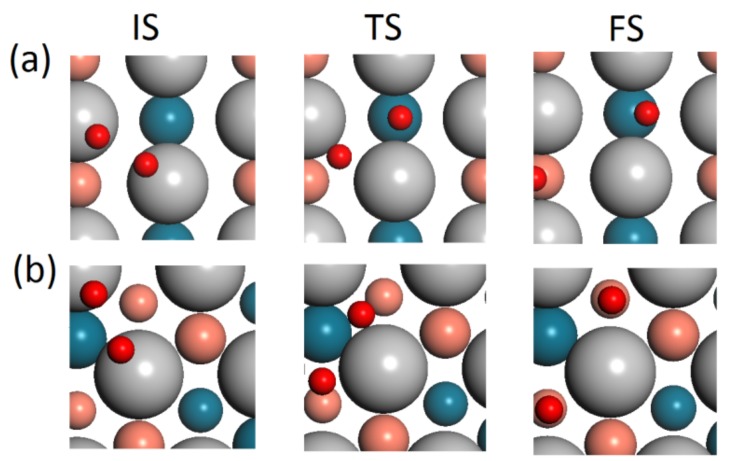
The top view of selected states along the path of O_2_ dissociation for (**a**) intermetallic Pd–Cu Pd/Pd–Cu(110) and (**b**) Pd–Cu FCC alloy Pd/Pd–Cu(111).

For the adsorption of O_2_, our previous paper and other studies have pointed out that the top-bridge-top (tbt) site is more stable and favored on metal surfaces compared with top site [43,45]. Here, we have calculated the adsorption energies on both the tbt site and top site, which results in the same conclusion as previous reports. Thus, in this work, the oxygen (gas) is initially placed on the tbt site. The calculated results are shown in Table 2. Upon being adsorbed on Pd surface, the O–O bond length is almost the same value as the various Pd surfaces. However, the O_2_ adsorbed on Pt monolayer has a larger O–O bond length of up to 1.362 Å. The adsorption energies of O_2_ on various Pd surfaces also follow the linear trend with respect to *d*-band center (*ε_d_*). These chemisorbed O_2_ can serve as precursors for ORR. Generally, two possible reaction mechanisms for the surface reaction of chemisorbed O_2_ are direct dissociation and hydrogenation into OOH [51]. Here, we will first consider the direct dissociation mechanism as the next step to investigate the ORR activity, where the dissociative barrier (E_barrier_) is regarded as an indicator to ORR activity. We will discuss the possibility of hydrogenation into OOH in the next section. The initial state (IS) is O_2_ chemisorbed on Pd surface, and the final state (FS) is two separated oxygen atoms occupied on hollow site. The nudged elastic band (NEB) method was performed to search for the transition state (TS) and the energy barrier of dissociation [44]. The top view of selected states along the dissociation path are shown in Figure 4. It is clearly seen that the O_2_ precursor will rotate from tbt site at the beginning to reach the transition state, where the two oxygen atoms are stretched to opposite direction gradually. The distance will continue to be enlarged until reaching the final state. As shown in Table 2, O_2_ chemisorbed on Pd monolayer on top of intermetallic Pd–Cu can be easily dissociated with energy barrier as low as 0.60 eV, while the dissociation of O_2_ on Pt monolayer on top of intermetallic Pd–Cu is unfavorable at room temperature with an energy barrier as high as 1.29 eV. It is noted that the energy barrier for Pd/Pd–Cu(110) is smaller than that of Pd/Pd–Cu(111), which can serve as evidence of enhanced ORR activity for intermetallic observed experimentally [32].

In order to figure out the universal trend and the design rule for ORR, we have plotted the well-known Brǿnsted–Evans–Polanyi (BEP) relations as shown in Figure 3b. We have added the data of Pt(111) for better understanding [45]. A linear relationship between the energy barrier (E_barrier_) and reaction energy difference (ΔE = E_FS_ − E_IS_) is observed. This linear correlation is the direct evidence that intermetallic Pd–Cu with Pd monolayer can serve as an alternate cathode material for fuel cell applications. The fitted line for oxygen dissociation is E_barrier_ = 0.714 × ΔE + 1.479. We can see that larger reaction energy difference can result in smaller energy barrier. The reaction energy difference is related to both adsorption energy for reactants and products. This is why too weak or too strong adsorption is not suitable for heterogeneous catalysis, which is also known as Sabatier principle [50].

### 3.3. The Potential Pathway of ORR on Intermetallic Pd/Pd–Cu(110)

Since intermetallic Pd–Cu is very promising for fuel cell applications, we will then explore the thermodynamics of the cathode reaction for intermetallic Pd–Cu. Generally, the cathode reaction is processed as follows: the adsorbed oxygen will react with H^+^ species into H_2_O.

1/2 O_2_ + 2(H^+^ + e^−^)→H_2_O

As discussed in the previous section, the two possible reaction pathways for surface reaction of O_2_ are direct dissociation or hydrogenation into OOH species. Here, we will explore the possibility of hydrogenation into OOH by considering the reaction energy difference (ΔE = E_FS_ − E_IS_). The results are summarized in Table 3. We can see that the possibility of hydrogenation into OOH is extremely low due to thermodynamically unfavorable. Besides this, the adsorbed OOH species may directly dissociate into O + OH on the catalyst surface.

Next, in order to investigate a complete cathode reaction pathway, we will investigate two possible reaction pathways as represented by reaction pathway I and reaction pathway II, where * denotes a vacant site on the surface.

Reaction pathway I

1/2O_2_ + *→O* (1a)

O* + H^+^ + e^−^→OH* (2a)

OH* + H^+^ + e^−^→H_2_O (3a)

Reaction pathway II

O_2_ + *→O_2_* (1b)

O_2_*+ H^+^ + e^−^→OOH* (2b)

OOH* + H^+^ + e^−^→O* + H_2_O (3b)

O* + H^+^ + e^−^→OH* (4b)

OH* + H^+^ + e^−^→H_2_O (5b)

**Figure 5:**
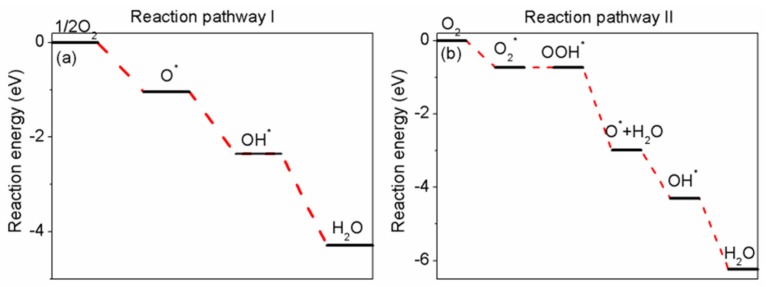
The reaction energy diagram on Pd/Pd–Cu(110) surface of (**a**) reaction pathway I and (**b**) reaction pathway II.

We have plotted the reaction energy diagram for the two reaction pathways in Figure 5. For the final step, the adsorption energy of H_2_O is only −0.13 eV. H_2_O molecule can diffuse freely from the surface. The surface is therefore ready to perform the next catalytic cycle. It is noted that, for reaction pathway II, hydrogenation to OOH (step 2b) is energetically unfavorable. However, the overall reaction is favorable.

**Figure 6:**
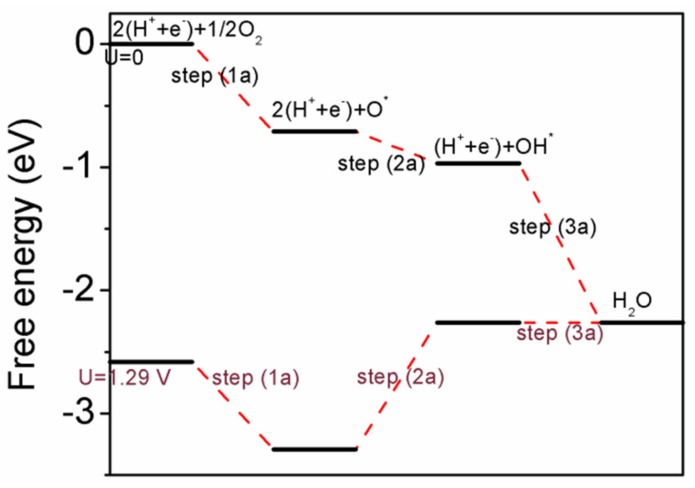
The free energy diagram for oxygen reduction on Pd/Pd–Cu(110) surface. It is noted that if the potential is higher than 1.29 V, step (2a) and step (3a) will become uphill.

We will then focus on reaction path I and calculate the free energies of the intermediates by the formula ΔG = ΔE + ΔG_U_ + ΔZPE − TΔS [52], where ΔE is the reaction energy from DFT calculations of adsorbed reactants or intermediates; ΔG_U_ (equal to −eU, U is the electrode potential) is the relevant bias effect due to electron and proton transfer; ΔZPE and ΔS are the zero point energy difference and the entropy difference between the adsorbed state and the gas phase, respectively; and T is the system temperature (T = 298.15 K in this work). The zero-point energy difference is obtained by DFT calculations of vibration frequencies of adsorbates. We have set the pH value to be zero. At a pH different from zero, we can correct the free energy by adding the free energy contributions due to variations in H^+^ concentration ΔG_pH_. The chemical potential of a proton/electron (H^+^ + e^−^) is equal to half of that of a gas-phase H_2_. The hydrogen atom is with reference to half of gas-phase H_2_; the oxygen atom is with reference to half of gas-phase O_2_. The Gibbs free energy diagram is shown in Figure 6, where we have considered potential effect. When the potential equals to 1.29 V, the connecting line for step (3a) is horizontal. Higher than this critical potential value (1.29 V), both step (2a) and step (3a) will become uphill.

## 4. Conclusions

With first principle calculations, we have investigated the potential of the intermetallic Pd–Cu alloy as electrocatalyst for ORR and the corresponding reaction mechanisms. On the basis of *d*-band model, we have calculated the adsorption energies (E_ad_) on Pd monolayer on top of various cores. The stronger adsorption strength on Pd/Pd–Cu(110) is attributed to both strain effect due to lattice mismatch and charge transfer from the substrate. We have then evaluated the oxygen dissociation barrier (E_barrier_) as an indicator to ORR activity by the nudged elastic band (NEB) method. The calculated barrier increases, following the order Pd/Pd–Cu(110) < Pt(111) < Pd/Pd–Cu(111). By plotting the Brǿnsted–Evans–Polanyi (BEP) relations, the intermetallic Pd–Cu with atomic cover layer Pd is found to have promising adsorbate–surface interaction. The proposed pathway for ORR indicates that adsorbed oxygen will go through direct dissociation mechanism rather than hydrogenation to OOH species. The following pathways, including O* + H^+^ + e^−^→OH* and OH* + H^+^ + e^−^→H_2_O, are found to be thermodynamically favorable. With these results, it is believed that the intermetallic Pd–Cu alloy with cover layer Pd may be an attractive alternate cathode material.

## Figures and Tables

**Table 1:** The calculated lattice constant for Pd–Cu alloy and intermetallic. The added data are from Reference [42] (density functional theory (DFT)) and Reference [41] (Exp.), respectively. Note that Pd–Cu alloy has face-centered cubic (FCC) structure while intermetallic Pd–Cu has B2 structure.

Composition	Structure	Method	Lattice Constant a = b = c (Å)
Pd	FCC	This work	3.949
		DFT	3.957
		Exp.	3.890
Cu	FCC	This work	3.633
		DFT	3.636
		Exp.	3.615
PdCu	B2	This work	3.021
		DFT	3.026
		Exp.	2.958
PdCu	FCC	This work	3.811
		DFT	3.816
		Exp.	3.760

**Table 2:** The calculated *d*-band center (*ε_d_*), adsorption energies of atomic O (E_O_) and O_2_ (E_O2_) and key parameters for O_2_ dissociation process including bond length, activation energy (ΔE) and energy barrier (E_barrier_). Note that the energy of atomic oxygen is defined as the half of that of O_2_. For O_2_ dissociation process, the initial state (IS) corresponds to the most stable O_2_ adsorption, the final states (FS) corresponds to two separate atomic oxygen atoms adsorption and the transition state (TS) is obtained by NEB calculations. We have added the data of Pt(111) from Reference [16,45].

Composition	*d*-Band Center (eV)	Adsorption Energy			Bond Length O–O (Å)	O_2_ Dissociation (eV)	
		E_O_ (eV)		E_O2_ (eV)			
		Hollow	Top	tbt		ΔE = E_FS_ − E_IS_	E_barrier_ = E_TS_ − E_IS_
Pt/Pd–Cu(110)	−2.66	−0.63	Unstable	−0.95	1.362	−0.36	1.29
Pd/Pd–Cu(111)	−2.12	−1.02	0.26	−0.36	1.341	−0.77	0.82
Pd/Pd–Cu(110)	−1.93	−1.18	0.31	−0.73	1.340	−1.34	0.60
Pd(111)	−1.86	−1.23	-	−1.18	1.342	−0.96	0.70
Pt(111)^Reference^	−2.19	−0.85	-	−0.62	-	−0.86	0.77

**Table 3:** The calculated reaction energy difference (ΔE) for adsorbed O_2_ hydrogenation to OOH. It can be seen that OOH species may dissociate into O + OH. The reaction energy difference is calculated by ΔE = E_FS_ − E_IS_.

Reaction Step	Reaction Energy Difference ΔE (eV)
O_2_* + H^+^ + e^−^→OOH*	−0.001
OOH*→O* + OH*	−0.011
